# Illinois State Dental Society

**Published:** 1888-06

**Authors:** C. N. Johnson


### ILLINOIS STATE DENTAL SOCIETY.

### TWENTY-FOURTH ANNUAL MEETING.

REPORTED FOR THE INDEPENDENT PRACTITIONER BY C. N. JOHNSON, L. D. S., D. D. S.

The twenty-fourth annual meeting of the Illinois State Dental Society was held in the Opera House, Cairo, May 8–11, 1888. The attendance was probably not so large as on some previous occasions, but in point of interest the meeting was a most decided success. Seldom have there been presented better papers, or more animated discussions elicited, while the best of good-fellowship existed throughout. All possible arrangements were made for the convenience of the members, and to Dr. J. J. Jennelle, of Cairo, great credit was due for the accommodations enjoyed. The Illinois Society has an excellent record as a working, energetic body, and this year's meeting will certainly add to its reputation in this respect.

### TUESDAY MORNING SESSION.

After the routine business of opening the meeting, the President, Dr. C. B. Rohland, of Alton, read his address.

He said it was the first time the society had held a meeting south of Jacksonville. He believed it was a timely move to come down into the southern end of the State, from the fact that dental societies are always followed by an increased interest in dental topics, not alone by dentists themselves, but by the general public. None but those who live in a community where ignorance concerning the teeth is prevalent, can appreciate the importance of employing every means of stimulating the public to a better understanding of their needs in this regard. They should be educated till they demand good dentistry, and then quackery will die for lack of support. This education should begin at home; that is, in each individual practice. It is not enough to simply perform an operation; we should teach the patient how to prevent a recurrence of the trouble by proper care of the teeth. Besides this, we may deliver lectures on the subject in our public schools. If a practitioner has not the literary accomplishment for this, let him publish in the local papers reprints of articles relating to dental hygiene. The papers will usually be glad to do so. In the rising generation lies our hope. Short lessons on this subject should be printed in our primary school books. The writer hoped for practical results from the forthcoming report of the committee appointed by the American Dental Association to consider this question.

During the past year two important events relating to the profession have occurred. One was the recognition of dentistry by the American Medical Association, and the other the formation of the Dental Section in the Ninth International Medical Congress. Without wishing to harp on the old question as to the relation between dentistry and medicine, he hoped the day would come when the title of D. D. S. would be as much honored as that of A. M. or M. D. Even now, we practically subscribe to the same code of ethics in our societies. The Dental Section of the Congress was just successful enough to show what might have been done by concerted action on the part of the whole profession.

Referring to the action of the First District Dental Society of New York regarding the suits of the International Tooth Crown Company, he said it was a good movement, but that the expenses should not fall entirely upon a few gentlemen; they should be borne by the profession at large. The lessons of Josiah Bacon's time should not be forgotten.

Another question he would like to see brought up, was the agitation of the matter relating to the exemption of dentists from jury duty. They hold nearly the same relation to the public as medical men, and should be granted the same privileges.

AFTERNOON SESSION.

Dr. A. W. Harlan offered the following resolution, which was unanimously adopted :—

WHEREAS, The majority of the dental colleges in the United States do not require of their students a longer period of pupilage than two years prior to the candidate's coming up for graduation, and—

WHEREAS, The Illinois State Dental Society believes that the time spent in college or in the study of dentistry is too short for proper preparation for entrance upon the practice of dentistry, therefore be it—

*Resolved,* That the National Association of Dental Faculties be requested at their forthcoming meeting in Louisville to adopt as a requirement for graduation that the student shall have studied three full years, including attendance on two full courses of college instruction in separate years.

The report of the Committee on Dental Science and Literature was then read by Dr. Louis Ottofy. The following is a summary: Are we scientific in what we do as well as in what we say? Is there harmony between our theories and our practice? Not altogether. We have not reached perfection, but we are slowly advancing. We are saving more teeth to-day than ever before. In this connection the crowning of roots comes to our aid. Two years ago this committee pointed out the need of some better method of preparing roots for bands, to obtain perfect adaptation. The same need is present to-day, No one has given us a satisfactory method, though many articles have been written upon it, the most of which make the operation appear very simple. We cannot be sure that the upper edge of the band fits the root properly, even if there is good adaptation at the lower edge. Hints have been given that a tool was forthcoming which would render this operation easy and certain of accomplishment, but up to date none is within reach. Let some one strive to solve the problem.

In the INDEPENDENT PRACTITIONER for November, 1887, Dr. William H. Trueman published an essay on the "Dental Pulp," in which he advanced some new arguments. He says: "In cases where the cavity approaches the pulp-chamber, the possibility or probability of inserting the filling without exciting serious irritation in the organ it contains has not seemed to me the most important question. That, in most cases, with ordinary care, is readily accomplished. The real question has seemed to be this: Can we, by so doing, secure the best results?" He admits the value of a vital pulp, but argues that it should be sacrificed if, by its retention, the permanency of the filling would be rendered doubtful, on account of imperfect preparation of the cavity through fear of encroaching too much upon the pulp, or from the bulk of the pulp-capping material when in place. He would not keep a pulp alive unless it were comfortable and well protected. His conclusions are summed up in the following, with which the writer agrees: "Much as I value a living pulp, I recognize that there are cases in which devitalization is necessary, preparatory to the insertion of a reliable preservative filling; that it is necessary in some cases where, could we avoid the mechanical difficulties encountered in securing the filling and satisfy the conditions deemed necessary to arrest decay, the salvation of the pulp would be reasonably assured. There are other cases where, from the condition of the pulp, its devitalization is merely a question of time, and practically the question is narrowed down to this: Shall we apply arsenic and devitalize in a few hours, or shall we attempt to preserve its vitality, with the almost positive assurance that the same result will be reached within two or three years?" This naturally leads to the question of immediate root-filling, which has been so much discussed of late. The truly scientific practice will likely be found in the middle ground between immediate filling and over-treatment.

Relating to histological subjects, the appearance of Dr. Black's articles on the periosteum and peridental membrane in book form is, perhaps, the most conspicuous work of the year. Dr. Stowell, in his "Microscopic Structure of a Human Tooth," has placed before the student a clear and concise representation of the dental tissues.

Regarding implantation, there is nothing new, except the report of Drs. Bödecker and Heitzmann before the First District Dental Society of New York. They examined a tooth that had been implanted six months, and found no "revivification of the dentine, cementum or pericementum." Implanted teeth are probably held in position mechanically. The writer reported eighteen cases in his own practice. Of these, three were unsuitable, leaving fifteen from which to judge. Twelve are firm, while three have failed, making a percentage of eighty per cent. successful cases. All except five have been implanted one year and over.

The germ theory of disease still claims the attention of the dentist. In this connection the question may be asked: What good is coming to the profession through knowledge possessed in regard to micro-organisms? In answer, it may be stated that we have gained valuable ideas through agitation of this subject. We treat many cases much more intelligently than before, but we need further light on this subject.

In the section of the report devoted to literature, special stress was laid on the importance of ceaseless study and active contribution to all our dental publications. Dentists are too lax about writing for journals. The lawyer and minister are almost of necessity men of letters, and there is no reason why the dentist should not have the same literary accomplishments. Jot down important items in practice, and finally write papers from them.

DISCUSSION.

*Dr. T. W. Brophy*—I would have been glad had the report told us how we could secure better adaptation of bands to roots. I have not seen proper instruments for this purpose. Roots are sometimes scooped out on the sides and so much contracted at the necks that, if made cylindrical, it would weaken the root. It might be better to have the band of some malleable material that could be burnished down closely to the surface.

I do not indorse immediate root-filling. We can never know that incipient abscess is not present, and if it is, trouble will always follow such an operation. It is to be deplored that this practice is advocated to such an extent in our periodical literature, on account of the danger of leading young men astray. We should be more conservative in our practice. A root may be filled immediately if we have destroyed and removed the pulp ourselves; but even in those cases, I would prefer to leave it a day or two after removal, in order to destroy any living tissue that might remain in the canal. For this purpose I know of nothing better than carbolic acid, ninety-five per cent.

*Dr. A. W. Harlan*—The report was conspicuous for what it omitted. There was no reference to the recent publications of Drs. Talbot, Haskell, Essig, Mitchell and others. It is the duty of the committee to search for and point out to the society that which has been accomplished in the past year toward the progress of dental science and literature. It is no part of their business to discuss methods of practice.

*Dr. E. Noyes*—The criticism is just, but I think this report comes nearer to the point than any previous one. The report suggests the question as to how much the theories have done in a practical way for our success. I think they have done very much. For many years we have been doing empirically what we now do scientifically, and the advantage of the latter course is that we are more likely to treat a case intelligently, and obtain good results with more regularity, than when we were in the habit of putting creosote into an abscess simply because we felt it would cure it, without knowing why it did so. In the whole line of treatment where pus infection plays a part, we have found that the tissues respond more promptly to our methods, and we can forecast with greater certainty whether or not a painful condition can be averted, than before our recent theories were brought forward. I think all our methods have advanced in consequence of theoretical teaching.

*Dr. Geo. H. Cushing*—I am pleased with that portion of the report which urges practitioners to contribute more liberally to the periodical literature of the profession. I would strongly advocate the keeping of notes in daily practice. From these, papers can be prepared with facility.

*Dr. A. W. Freeman*—No practitioner can get along without four or five dental journals. In addition to this, he should read those books on dental topics which are most suited to his wants. I think the committee should point out the characteristics of the various books published from year to year, so that the members could select those which, individually, seemed most desirable for them to obtain, as it is too expensive for the average dentist to purchase all that are brought out.

*Dr. Geo. D. Sitherwood*—I take a number of dental journals, but so many reprints are made that I find little new material in them. I think more original matter should be published.*

*Dr. G. V. Black*—I would suggest that the gentlemen who criticise the journals for not publishing much original matter should write for the periodicals more extensively themselves.

*Dr. J. W. Wassall*—Dr. Freeman thinks it is too expensive to buy all the dental works published. I believe it is the best invest-

---

* The INDEPENDENT PRACTITIONER does not feel that it is amenable to criticism on this score. Not a line which appears in it can ever be found elsewhere, unless copied from it—always, of course, excepting items of news, which are common property.—EDITOR.

ment a dentist can make. When you buy books you buy brains, and you can no more afford to do without them than you can afford to economize in instruments.

*Dr. J. J. R. Patrick*—This defect of not having better original matter in our dental journals is more the fault of the dentists than the journals. When a periodical is published by a dental supply house, the latter feels under an obligation to its patrons, and will publish anything sent in by them. Another fault with societies is that they permit dental supply houses to publish their programmes and orders of business. They do not seem to recognize the fact that they pay for this in the end; it is a system of indirect taxation. The society ought to pay all its own expenses, and not rely on any supply house.

On motion the subject was passed.

The next order was the report of the Committee on Dental Arts and Inventions, by Dr. J. Frank Marriner.

Among new appliances and methods that have come to our notice during the past year, the following are mentioned. Colson's Plugger. Dr. Newkirk's method of mounting wood polishing points. A new engine mallet, of small size, with stroke similar to electric mallet, with the advantage of instantaneous change by a thumbscrew; the blow can be made stronger than the Bonwill or electric mallet (E. E. Cady, *Dental Review*, Feb., 1888). Dr. W. G. A. Bonwill's method of packing amalgam by laying Japanese bibulous paper over small pieces of amalgam in the cavity, and pressing or burnishing them into position, repeating until the operation is complete; the committee believes that those who once try it will not abandon its use. A rubber-dam clamp, with double hooks or ears to prevent slipping down too far on the neck of the tooth, and crowding the gum (Independent Practitioner, 1887, page 665). Improved labial or buccal clamps, by Dr. D. B. Freeman, Chicago; also by the same gentleman, new forms of matrices. A circular description of all Dr. Freeman's late inventions may be had on application, and the committee recommends an investigation. The Gould Dental Chair, by Charles A. Rigdon, Warsaw, Ind., is also thought worthy of notice.

Two whole mornings—Wednesday and Thursday—were devoted to clinics and exhibition of new appliances. This will be considered further on in the report.

WEDNESDAY AFTERNOON.

Dr. John J. R. Patrick read a paper on "Dental Morphology and the Etiology of Irregularities." In the development of different parts of the body all morphological and physiological changes are identical with those which commenced the life history of the animal. All organized beings must develop from simple cells, which, in order to be productive, must meet and blend with dissimilar cells. The result of this blending is a true parent cell, which cells become a germ, the germ an embryo, the embryo a fœtus, the fœtus a child, and the child an adult. First, in the formative cell, we have a nucleus; then segmentation begins; then two cellular membranes are developed, the epiblast and hypoblast. Later on, a third membrane appears between these two, and is called the mesoblast. It is supposed to be derived from the hypoblast. These three membranes form the germ area or gastrodisk. The epiblast gives rise to all the epidermis, the epithelial lining of the cerebro-spinal canal, the ventricles of the brain, the cerebro-spinal nervous centers, and various parts of the organs of special sense. The hypoblast is the source of the epithelium in the alimentary canal, while the mesoblast gives rise to the remaining parts of the body, as the dermis, muscles, bones, connective tissue, blood vessels, kidneys, urinary apparatus, etc. Now it is difficult to understand how a tooth should be endowed with these three—the epiblast, hypoblast and mesoblast—when the latter alone contains all the organs of construction necessary for the production of teeth, bones, muscles, connective tissue, etc. Surely, a membrane with so much constructive ability could produce a tooth without aid. There is one thing certain, however; the fertilized cell is different from either the maternal or paternal cells, because it is in the real sense the male ancestor and female ancestor of all the numerous generations of cells which afterwards build up the many celled organism. This truth is verified by the fact that the child inherits many characteristics of both parents. We see the large teeth of one parent and small jaw of the other in the same child, and also the small teeth of one parent and large jaw of the other in another child.

In studying the development of the teeth, if we take a young hog between eight and ten months old, we find the homologue of a child's first permanent molar fully developed. If we remove the lower jaw, zygoma and malar process, we expose a long, puffy ridge, running from the first permanent molar backward and upward, to what in time becomes the tuberosity. Remove the outer thin plate of the puffy ridge, and you will see the two cells of the last permanent molars in their saccular stage of development. Each sac enveloping the future tooth is connected with the gum by a funicle, which is merely an elongation of the sac. It is in the nature of an umbilicus, and the sac represents the placenta. This passes through the same developmental phenomena as does the mammalian embryo.

In the development of the sac, the funicle enlarges at the deep end, becomes detatched from the gum, and contracts upon the newly formed enamel. At the same time, nutrient vessels from the surrounding alveola emerge and connect with the sac, thus establishing mutual intercourse of circulation. The enamel of the tooth being completed, the crown emerges from the alveolus, and the sac gradually dips back to the neck of the forming tooth. It there remains stationary, and the cementum, which some time previously began to form at the neck, continues to grow in the form of hollow cylinders, enclosing the granular protoplasm, which eventually is productive of dentine.

During all the developmental processes, the sac is the formative membrane of the enamel and cementum, so that in a morphological point of view the relations of the cementum and enamel show them to be homologous. Thus it appears that the line of succession in the formation of the hard tissues of the tooth is enamel, cementum and dentine.

Prof. Flower's diagrams of nerves depict the superior maxillary division of the fifth, with the posterior and anterior dental branches throwing off filaments direct to each particular tooth root. This diagram may be true regarding the main branches, but the branchlets of these branches, making direct connection with the roots, cannot be shown by dissection. I regard the diagram as being conventional anatomy in this respect. The teeth of the inferior maxilla are more directly connected with the inferior dental vessels and nerves, than are the teeth of the superior maxilla with the corresponding vessels and nerves of that region. This is due more to a restriction of territory than to necessity, and it is this cause which prevents the development of supernumerary teeth in the lower jaw. They seldom appear in the inferior maxilla, because there is too little room in this region to permit of such extravagance. Superdentition is therefore much more common in the upper jaw.

Examples of supernumerary teeth are not always anomalous in form, and rarely make connection with the superior dental vessels and nerves. They usually obtain their supply from the surrounding tissue. They are sometimes found imbedded in the palate process, with the crown directed toward the alveole, and the question naturally arises; How can such teeth form direct connection with the superior dental nerves ? Yet they have obtained from their surroundings nutriment sufficient to develop enamel, cementum and dentine.

The deciduous teeth are further evidence to establish the fact that the tooth germ can develop its several parts independent of special nerves and arteries, for these teeth are supplied by deciduous vessels, surrounded by deciduous bone, and are exuviated in course of time, as useless material. A study of the interruption of tooth development at certain stages gives useful information as to the series of changes by which the perfect form is evolved. The essayist cited a case in illustration in which the inferior right second permanent molar passed little beyond the saccular stage, the enamel and cementum being formed, but little or no dentine. It caused no pain, though much decayed, and was sore and loose. It had no roots, but was larger than the adjoining teeth, and much below them. It was hollow and spherical, and was made up of cementum and enamel. It is evident that had this tooth remained in the alveolus, its connection with the gum severed, and no other source of supply than the nutrient vessels from the alveolus, which would be sufficient only for the development of the cementum, that the granular mass of protoplasm receiving no nourishment would degenerate, and in all probability produce a dentigerous cyst.

DISCUSSION.

*Dr. G. V. Black*—I am puzzled to know how to open this discussion. I have looked over Dr. Patrick's paper, and I will probably find it necessary to disagree with him. We have not looked at the subject from a similar standpoint, and the same landscape may appear differently when viewed from different or opposing aspects. His general description of the ovum and its segmentation is in the main correct, but it is not certain that the kidneys and urinary apparatus come from the mesoblast. He is probably right in saying that the tooth will be developed without connection with the nervous system, for we find that in some malformations no nervous system is present, and yet the child may grow nearly to term, and have teeth developed in accordance with its age. It is possible, then, that some teeth may grow without nerve development, but we generally find that these teeth are sensitive.

My friend has taken a view of the subject which surprises me somewhat, and yet macroscopic specimens, especially those preserved in alcohol, present very much the view that he has given us. You will find the epithelial cord, in macroscopic examinations, presenting the appearance he has described, and I have not found any vessels in it; but when it breaks up we will find vessels about it. (The speaker here illustrated the point on the blackboard.) The careless pulling of tissues apart is responsible for much error in judgment; they should be carefully cut *in situ*. We will find connection with blood vessels in different directions from those indicated by the essayist.

There are two other points on which I take issue with Dr. Patrick. One is that the enamel organ arises from the epiblast, not the mesoblast; the other, that dentine has generally begun to form before there is any cementum. As to the matter of absorption, which the essayist mentioned in his supplemental remarks on his paper as being a visionary theory, I will say that it is not in any degree a supposition. We may trace it in the hard tissues, as bones and teeth, and we know that a certain portion is being dissolved. It is the way in which the roots of temporary teeth get away, and also the roots of implanted teeth, and those membranes which are no longer useful in the development of teeth are removed in the same manner. The new thing to be done is done by new tissue, developed for the purpose.

Now as a tooth crown is developed, the enamel organ grows thinner and thinner, and finally disappears, and in the case of impacted teeth the enamel presents itself against the connective tissue in an abnormal relation, and hence in these cases we are liable to get cysts, abscesses, etc. The impacted tooth causes irritation, usually about the time of the formation of its roots, when it is thrust forward against these tissues. The essayist drew attention to the fact that there was no dentine in the specimen he describes; but I have always found dentine present in deformed teeth, by making sections from them.

Membranes, such as the mucous, synovial, etc., are usually supports for a functioning tissue, as the epithelium which covers them. A capillary is a capillary by virtue of its pseudo-epithelium. (I use the word pseudo to represent the thelia developed from the mesoblast, in contradistinction to that from the epiblast and hypoblast.) I have found supernumerary teeth in the lower jaw, but they are very much more common in the upper jaw. I once saw a young lady with five incisors on the lower jaw, all in line, and I have also seen four inferior molars on either side, in the same mouth.

*Dr. Carroll*—I would like to ask Dr. Black if he regards the supernumerary teeth as mere accidental abnormalities, or an effort at a return to previous types.

*Dr. Black*—I regard them as mere vagaries. We find monstrosities in all forms of nature. We often see little buds growing from the epithelial cord, and the supernumerary teeth likely come from them. It is hardly possible to prove, but theoretically it seems probable. These teeth are generally deformed, but sometimes we get perfectly proportioned teeth, and this often leads to the careless extraction of a normal tooth instead of the supernumerary one.

*Dr. Taylor*—Why do these occur in the second dentition, and not in the first ?

*Dr. Black*—It is difficult to tell why, but they do present themselves sometimes, even in the first dentition. I once saw an extra lateral incisor in a child four years of age.

*Dr. Taylor*—Will teeth be perfect in their formation when there is no direct nerve connection ?

*Dr. Black*—I believe they will. I have seen a fœtus where the nerves were absent, and yet the teeth were developed to term perfectly.

*Dr. Patrick*—I purposely avoided the question just raised by Dr. Black in his remarks, because in matters of physics I deal as little as possible in mere belief and speculation. The past history of scientific research shows that nothing has done so much to retard progress as the interference of beliefs and speculations, especially when promulgated by ingenious men. But since this matter has been mentioned, I will give my idea about it.

In the drawing made by Dr. Black, there was a representation that the tooth dips down through the epidermis into the rete mucosum, and that there the enamel organ is met by a distinct projection of this deep-seated portion, and into which, by a system of invagination, it passes up into these enamel cells from the dermis. So it appears, according to this, that the tooth is made up of the epidermis, and the dermis—the epithelium and the rete mucosum —the epithelium dipping down to the rete mucosum, and the rete mucosum rising in the form of a papilla, projecting into the enamel organ. Now this papilla, arising from the deep-seated portion of the mucous membrane, which is said to be the dentine organ, is of course supplied with nutrient vessels. Yet we find the tooth is in a saccular stage before the roots are formed; that the dentine organ is a granular mass of protoplasm without vessels— or at least if the vessels are there they are not filled with blood, there being no connection at this portion of the sac with surrounding tissue—for while the fœtal tooth is in this saccular condition it receives its nutriment by way of the funicle or umbilicus, and later from the cell wall. According to the drawing on the board, the sac would have to rise from the base of the papilla and envelop the enamel organ, make connection with the cord or funicle, and then be detached with the cord when the enamel passed through the sac. Now we do not find any such condition, and I defy any person to prove it.

Subject passed.

Quincy was selected as the next place of meeting.

The society honored itself in electing the following officers for the ensuing year :

*President*—Dr. Geo. H. Cushing, Chicago.

*Vice-President*—Dr. J. J. Jennelle, Cairo.

*Secretary*—Dr. Garrett Newkirk, Chicago.

*Treasurer*—Dr. T. W. Pritchett, Whitehall.

*Librarian*—Dr. W. B. Ames, Chicago.

*Executive Committee*—Drs. P. J. Kester, J. W. Cormany and J. W. Wassall.

(TO BE CONTINUED.)

